# Menopausal Women's Health Care Method Based on Computer Nursing Diagnosis Intelligent System

**DOI:** 10.1155/2021/4963361

**Published:** 2021-07-24

**Authors:** Qing Chao, Weiping Ma, RuiJia Xu, Lingyan Wu, Youwen Zhang, Miao He, Ke Yang, Wanxia Yao, Rong Peng

**Affiliations:** ^1^The First Affiliated Hospital of Xi'an Jiaotong University, Xi'an, Shaanxi 710061, China; ^2^Ankang Central Hospital of Shaanxi Province, Ankang, Shaanxi 725000, China; ^3^Shenzhen Samii Medical Center, Shenzhen, Guangdong 518118, China; ^4^The Third People's Hospital of Shenzhen, Shenzhen, Guangdong 518118, China; ^5^HanZhong Central Hospital of Shaanxi Province, HanZhong, Shaanxi 723100, China; ^6^The Hospital of Shenzhen Technology University, Shenzhen, Guangdong 518118, China; ^7^Ankang Hospital of Traditional Chinese Medicine of Shaanxi Province, Ankang, Shaanxi 725000, China

## Abstract

Taking into account the current feature extraction speed and recognition effect of intelligent diagnosis of menopausal women's health care behavior, this paper proposes to use a cross-layer convolutional neural network to extract behavior features autonomously and use support vector machine multiclass behavior classifier to classify behavior. Compared with the feature images extracted by traditional methods, the behavioral features extracted in this paper are related to the individual menopausal women and have better semantic information, and the feature description ability in the time domain and the space domain has been enhanced. Through Matlab software, using the database established in this paper to compare its feature extraction time, test classification time, and final recognition accuracy with ordinary convolutional neural networks, it is concluded that the cross-layer CNN-SVM model can ensure the speed of feature extraction. It proves that the method in this paper can be applied to the behavioral intelligent diagnosis system for intelligently nursing menopausal women and has good practical value. This paper designs a home care bed intelligent monitoring system, which can automatically detect the posture of the care bed, and not only can change the posture of the bed under the control of personnel, but also can automatically complete the posture conversion according to the setting. At the same time, the system has the function of monitoring the physical condition of the person being cared for and can detect the heart rate, blood oxygen, and other physiological indicators of the bedridden person. In addition, the system can also provide a remote diagnosis function, allowing nursing staff to remotely view the current state of the nursing bed and the physical condition of the person. After testing, the system works stably, improves the automation and safety of the nursing bed control, and enriches the functions of the nursing bed.

## 1. Introduction

Menopausal syndrome is a common disease of menopausal people. Without obvious pathological factors, women have a continuous year of amenorrhea or permanent cessation of menstruation, which is called menopause [1]. Before and after menopause, due to low estrogen levels, the aging of the ovaries affects the function of the hypothalamus-pituitary-ovarian axis and causes menstrual disorders, hot flashes, sweating, irritability, depression, dizziness, fatigue, bone and joint muscle pain, and headaches [2]. Palpitations, skin abnormalities, and many other discomforts are the clinical manifestations of menopausal syndrome. Menopausal women are often accompanied by obvious psychological disorders, such as anxiety, depression, and other emotional and psychological reactions, which seriously affect the quality of life of middle-aged and elderly women [3]. A survey shows that about 70% of women will have psychological and physical discomfort, showing varying degrees of anxiety, depression, and other physical and mental discomfort symptoms, and only 30% of menopausal women undergo self-regulation without the manifestations of menopausal syndrome.

The pathophysiological mechanism of menopause is generally believed to be the imbalance of neurotransmitters, hormones, cytokines, etc., caused by the hypothalamic-pituitary-ovarian axis or adrenal dysfunction caused by the decrease of estrogen levels in the body [4]. In addition, studies have found that endocrine disorders are not the only cause of menopausal syndrome [5]. In addition, it is also related to multiple factors such as family factors, cultural and social factors, psychological factors, and personality and behavior status. As the childbearing age increases, the number of ovarian follicles decreases. When the number of primordial follicles drops to an extremely low level (<1000), the key level of the feedback system will also decrease, and this series of endocrine changes occurs, disrupts the normal cyclical ovarian hormone secretion and ovulation, affects the highly coordinated interaction of ovarian hormones, pituitary gland and hypothalamus with normal menstrual cycle, and causes the hypothalamic-pituitary-ovarian axis to be confused. This damage involves the transition period of the normal ovulation cycle, until the woman's last menstrual period. In women during menopause, the number of follicles in the body is significantly reduced, and the remaining follicles are low in response to gonadotropin (Gn) or completely lose their response, so the frequency of ovulation is reduced [6].

Aiming at the speed of feature extraction and recognition effect in behavioral intelligence diagnosis, combined with the characteristics of menopausal women's behavior patterns, this paper proposes the use of cross-layer convolutional neural networks to autonomously extract features. Combined with support vector machine multiclass behavior classification of nursing object behavioral intelligence, diagnosis programs are designed from the aspects of network structure, activation function, classification mechanism, and parameter selection. This article shows the hardware objects of the home care bed intelligent monitoring system and the care bed monitoring center software and shows the test results of the posture detection and control functions, timing control functions, menopausal women's status monitoring functions, and remote diagnosis functions in the system. The results show that the designed system function has achieved the expected effect.

## 2. Related Work

Japan's Panasonic Corporation took the lead to release a nursing bed “Robotic Bed” that can be freely converted into a wheelchair [7]. The nursing bed is composed of an electric bed body and a wheelchair. The wheelchair is embedded in the bed body and can be automatically popped up or put away. When the wheelchair is retracted into a sitting posture, it can be separated from the bed body; when the wheelchair is unfolded, it can be fitted with the bed body to restore the bed surface. In addition to the function conversion of the bed and chair, the nursing bed is also designed with a motion unit at the bottom of the bed. In the wheelchair mode, menopausal women can detect people and obstacles to help users avoid obstacles; the ceiling display can be used as a home appliance controller and can realize remote diagnosis [8]. This kind of nursing bed has a novel design concept, simple use, and convenient operation, so that patients can basically take care of themselves. The German Volk Company has passed the balanced force and safety load-bearing test and produced a multifunctional nursing bed that can be adjusted in various positions, which provides a good guarantee for users in terms of safety and reliability [9]. In addition to ensuring the advantages of the electric bed itself, it effectively realizes the practicability of additional functions, such as detachable guardrails on both sides of the bed, an infusion stand placed along the bed, and a height-adjustable dining table. Scholars from Singapore Polytechnic Institute designed an FPGA-based intelligent low-cost nursing bed to prevent bedsores in a clinical environment [10]. When the bed surface temperature exceeds a predetermined threshold, an alarm will be issued to remind the nursing staff to help the patient turn over, which can effectively reduce the formation of bedsores.

The United States is also at the world's leading level in the field of intelligent nursing beds. The most representative ones are Stryker Medical Device Company and Hillrom Company [11]. The InTouch intelligent nursing bed developed by Stryker has an additional safety guardrail, and many intelligent elements are added on the basis of functions such as lifting and turning over [12]. For example, it supports 24 languages and can be used in different language countries. Based on this, the nursing staff can formulate a turnover schedule suitable for the user; it can record the user's weight change in real time, so that the nursing staff can adjust the nursing plan in time; it also has the functions of automatic cleaning and playing music to maximize the service to the user [13]. The price of this product is too high, and it is currently mainly used in high-end hospital wards. The Totalcare P500 intelligent nursing bed launched by Hillrom also realizes basic functions such as getting up and turning over. Its advantage is that it can realize intelligent processing. It collects the temperature, humidity, pressure, and other information of different parts of the user in real time and performs comprehensive processing. Operations such as automatic heat dissipation and turning over can effectively prevent bedsores; in addition, the product also has emergency functions such as one-button reset and one-button alarm to ensure user safety to the greatest extent [14].

The Swedish company ArjoHuntleigh has developed an Enterprise9000 nursing mattress with automatic cleaning and sterilization functions. In addition to realizing the basic functions of the nursing bed nursing mobile position, this model of nursing bed is equipped with a weight scale integrated in the nursing bed, which can continuously and accurately monitor the weight of the nursing staff and display the results [15]. In addition, the nursing bed supports automatic cardiopulmonary resuscitation function (CPR) and manual CPR functions on the left and right sides of the bed to respond to emergencies. The evaporative automatic cleaning and sterilization system carried by the nursing bed can be set to time and clean at designated points. The nursing bed ensures the cleanliness of the nursing bed and improves the comfort of the nursing staff [16].

Related scholars have developed a multifunctional nursing bed based on remote monitoring [17]. The development of the system adopts DirectShow, SQL Server, and Visual C++ programming to realize the physiological parameter collection, information data management, and audio and video monitoring of the care recipient, achieving a true comprehensive safety monitoring [18–20]. They use the developed monitoring system to display the collected physiological parameters of the person being cared for, such as heart rate and pulse on the system client, realizing monitoring at all times and storing the system that can be monitored by the system of the caring person in the system database [21, 22]. It is easy to analyze its health status.

## 3. Overall Scheme Design of Computer Nursing Diagnosis Intelligent System

### 3.1. Structure of Computer Nursing Diagnosis Intelligent System

**Figure 1:**
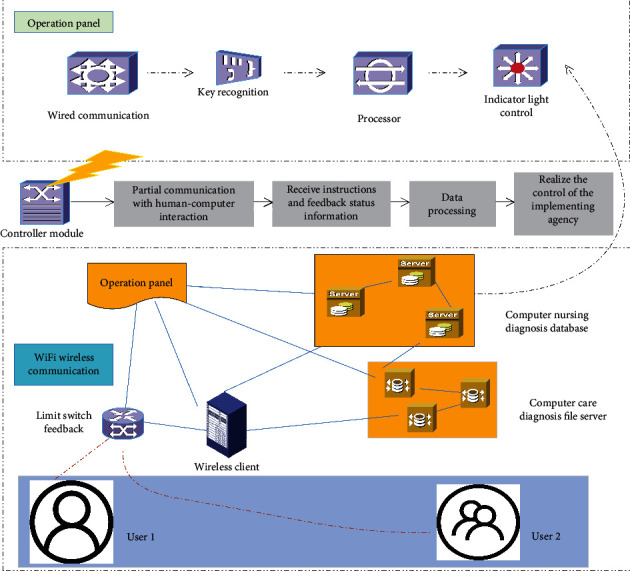
Control system structure.

The computer nursing diagnosis intelligent system is divided into several modules, and the modules are interconnected and cooperating with each other to realize the stable operation of the entire system, as shown in Figure 1. The core part is the controller module, which is responsible for communicating with the human-computer interaction part, receiving its instructions and feeding back status information, and processing data at the same time to realize the control of the actuator, which is an important part of the system. In order to facilitate the user to control the controller, the wireless interface is set up, and the application program is installed through the client terminal such as mobile phone and tablet, and then the human-computer interaction is completed through the application program. This determines that the wireless technology can only use technologies that are supported by clients such as general mobile phones and tablets.

The remote diagnosis part can control the operation of the nursing bed by controlling the controller through wireless communication. The current wireless technologies supported by mobile phones include Bluetooth and WiFi. The advantages of Bluetooth technology are low-power consumption and high security level, but the disadvantage is that the communication rate is lower than WiFi, the wireless transmission distance is also lower than WiFi, and Bluetooth communication is often a single-point pair. For single-point communication, WiFi communication can realize data sharing through wireless routing to achieve the purpose of accessing the Internet.

The nursing bed can also collect real time information through a camera and then connect to the Internet through a wired way. In this way, the information of multiple nursing beds can be viewed remotely without going to the front of the nursing bed. This is extremely convenient for medical staff who need to check the ward frequently. At the same time, the patient's family and friends can also check the patient's condition at any time within a certain authority. In the hospital, it is possible to design the basic information and health status of the patient filled in by the medical staff on the application, realize the “electronic medical record,” build a platform for patients and their families to understand the situation, and maximize the convenience of users.

### 3.2. Computer Hardware Platform of the Controller

For the client, it can be divided into two modules to realize two ways of wired control and wireless control, respectively. For the wired control method, an operation panel is set, the operation panel obtains power from the controller through a cable, and the processor is set to communicate with the controller. A human-computer interaction interface is set on it, and the user interacts with the user by setting function buttons and corresponding status indicators. The function buttons provide user instruction input, and the indicator lights give the user the system status. The wireless control method is to set up a WiFi module, which converts the signal of the controller into a WiFi signal through the WiFi module, communicates with the wireless router, and communicates with the mobile phone or computer through the wireless router, so as to realize the control target of the wireless client to the system. The various modules of the controller are interconnected and independent of each other. Different modules only interact through interfaces without coupling interference. This structure enhances the stability of the system and, at the same time, facilitates the expansion and development of the system.

**Figure 2:**
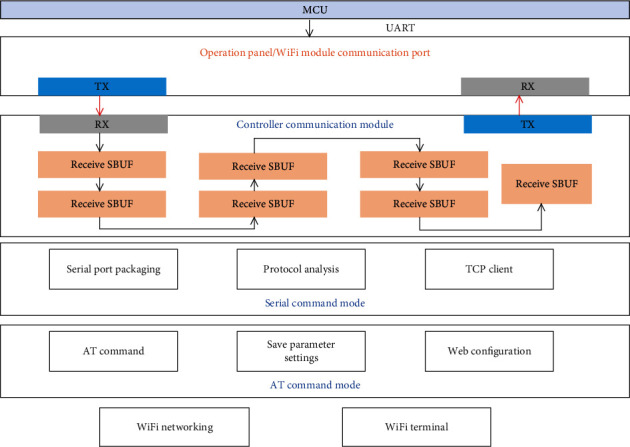
Structure diagram of communication module.

For a control system to work normally, even if it only needs to implement the simplest functions, it must have a stable and reliable power supply. The importance of the power supply module is self-evident. The control system uses an external switching power supply to realize the conversion from mains power to 24 V DC power supply. Considering the communication frequency of the system, etc., the switching power supply frequency is selected as 300 kHz. The switching power supply output 24 V DC power supply is divided into two ways: one way is converted to 5 V and 3.3 V DC power supply for other modules through the controller step-down conversion chip, and in the other way, power supply directly drives the worm gear DC motor. Therefore, the switching power supply needs not only a larger output power to drive the motor, but also a smaller output ripple to supply the power conversion chip. The structure diagram of the communication module is shown in Figure 2.

The Wi Fi module is an important module for wireless control. Through the WiFi module, the serial signal of the controller processor can be converted into a WiFi signal and sent to the wireless client, and at the same time, the wireless signal from the client can be converted into a serial signal to communicate with the processor. Users can connect to the wireless router through mobile terminals such as mobile phones, and then they can access the WiFi module that is also connected to the wireless router and then communicate with the controller. This allows users to interact with the control system extremely conveniently and, at the same time, connect the control information to the Internet, which facilitates networked management and information sharing.

According to requirements, the low-power serial port to WiFi module USR-WIFI232-SA is adopted, which can realize the transparent transmission of the signal from the serial port to the WiFi. One end of the serial port to WiFi module is a serial port signal, which can be compatible with the TTL level of the single-chip microcomputer. Through the built-in processing chip, the universal serial port communication protocol and the IEEE802.11g communication protocol can be converted mutually.

#### 3.2.1. Software Structure of the Controller

The hardware of the controller is only the basis of the platform, and the real logic functions need software programming to achieve. In order to make the software structure clear, reduce the degree of coupling, and facilitate expansion and maintenance, the software program is also modularized, and different functional modules are interconnected through interfaces and work independently of each other. The main control module is the central module that contacts each module, is responsible for the operation of the main flow of the program, and calls other functional modules to achieve the required functions and coordinate processing. The motor control module is responsible for receiving the signal input of the motor limit switch, judging the state of the motor and then the state of the nursing bed, so as to realize the logical control of the motor and the nursing bed. The function of the serial communication module is to be responsible for the dual-channel serial data communication between the operation panel and the wireless client, analyze the received data according to the specified communication protocol, and package the data to be sent according to the protocol. At the same time, it is responsible for data processing, including storing, adding, and deleting part of the data. The operation panel module realizes the analysis of the key input on the panel and the control of the indicator light by processing the data communicated with the operation panel. The control of the indicator light needs to contact the status of the motor and the cleaning box. The wireless client module is responsible for parsing the control commands of the wireless client, processing, and feeding back the functional status of the motor and the cleaning box.

**Figure 3:**
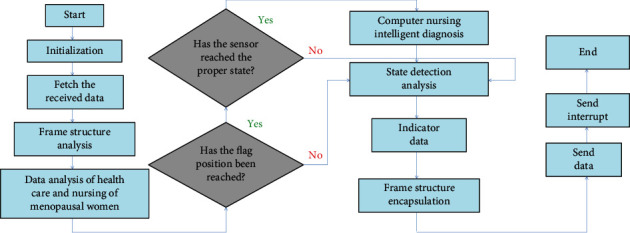
The main flow chart of the control program.

The main flow of the software is the main program loop detection, as shown in Figure 3. Keil is used as a programming tool to program and control the STC15 microcontroller to achieve the required control functions.

The program is initialized from the very beginning and then continues to perform loop detection. If there is an interruption, the protection site will jump to the interruption processing and continue to execute the main program in a loop after completion. The main loop fetches the data from the receiving buffer, then analyzes the frame structure to obtain specific and effective data, and then obtains the specific action instruction flag according to the data analysis. First, you check the motor action flag; if it is set, and the motor limit switch is detected to be in a proper position, then the corresponding motor action will be controlled. Secondly, you check the action flag of the cleaning function and output the cleaning box control signal if it is set. Then, you check the status input of the cleaning box and send out the control data obtained from the analysis to the indicator light.

When the main program is running, if there is an interruption, the scene will be protected first. When detecting that the interrupt source is a timer interrupt, the initial value is reloaded, and the function content is processed.

## 4. Improved Intelligent Diagnosis Method of Nursing Object Behavior Based on Convolutional Neural Network

### 4.1. Behavioral Intelligent Diagnosis Plan for Intelligent Care of Menopausal Women

For CNN, due to the nature of convolution and downsampling, the translation change of the image has almost no effect on the extracted feature vector, and it is not prone to overfitting. In addition, different convolutions, downsampling, and feature vectors can also be used to control the fitting ability of the entire model, so that when overfitting occurs, the feature dimension is reduced or when underfitting occurs, the size of the convolution kernel can be changed to increase the volume. Compared with other feature extraction methods, CNN-based feature extraction methods are more accurate and flexible.

**Figure 4:**
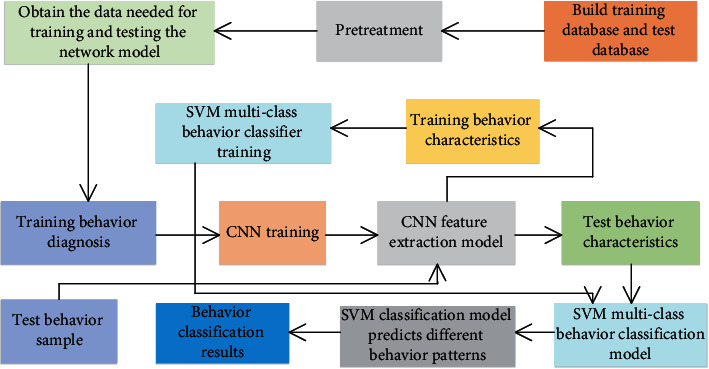
The flow chart of the behavioral intelligent diagnosis plan for nursing objects.

The choice of classifier is also extremely important. Currently, neural networks, HDR decision trees, and SVM classifiers are commonly used. SVM is a general learning algorithm with strong generalization ability and has a good classification effect in the field of image recognition. Compared with the commonly used Softmax classifiers, SVM has smaller generalization errors and is less prone to underfitting. It overcomes the overlearning or insufficient generalization ability caused by neural networks that are easily affected by structure and sample size. The data reflects good analytical ability and learning ability. In addition, effective features have been extracted through the convolutional neural network, and complex classification mechanisms are no longer needed in the later classification stage. In summary, the CNN feature extractor and SVM multiclass classifier are finally selected as the implementation methods of feature extraction and behavior classification and recognition.  Figure 4 shows the flow chart of the behavioral intelligent diagnosis program of nursing objects in this paper.

### 4.2. CNN Cross-Layer Structure Design

The cross-layer CNN-SVM model consists of 7 layers, which are 1 input layer I, 2 convolutional layers (C1, C3), 2 downsampling layers (S2, S4), 1 fully connected layer F5, and 1 output layer O. In particular, in order to effectively fuse high-level behavioral features with low-level behavioral features, this paper connects the first downsampling layer S2 across the convolutional layer C3 and downsampling layer S4 to the fully connected layer F5. Unlike the DeepID network, the DeepID network connects the last downsampling layer with the fully connected layer, and the connection weights need to be adjusted and learned. The cross-layer CNN-SVM model proposed in this paper directly connects the S2 layer to the fully connected layer and uses the output of S2 as part of the input of F5. That is, the fully connected layer F5 receives the input from the downsampling layers S2 and S4. The weight of the layer connection is fixed, and there is no need to readjust and learn.

The input layer is a 48 × 48 image, so the number of neurons in the input layer is 2304. The C1 layer is a convolutional layer that includes 6 feature maps. It is obtained by convolution operation on the input image by a convolution kernel with a size of 5 × 5, and the size of each feature map is 44 × 44. For each convolution feature map, there are 25 weight parameters *k* and a bias parameter *b*, and there are 6 convolution feature maps. There are two trainable parameters and 12615 neurons. The calculation formula is

(1) $$\begin{array}{c} \text{C}\left(1;i,j\right)=f\left[u\left(1;i,j\right)\right]=f\left[x_{i}\cdot k\left(1;i,j\right)-b\left(1;j\right)\right]. \end{array}$$

The S2 layer is a downsampling layer. The feature image output by the C1 layer is downsampling with a sampling unit of 2 × 2 using the maximum pooling method to obtain the same number of feature images, but the size is reduced to 22 × 22, a total of 2904 neurons. Due to the complexity of the cross-layer structure, in order to prevent the miscalculation of error partial derivatives when correcting parameters, we improved the calculation method of the down-sampling layer, simplifying the calculation process by removing the multiplicative bias and additional bias. To achieve the purpose of accurately calculating the backpropagation error and quickly correcting the training parameters, the calculation formula is

(2) $$\begin{array}{c} \text{S}\left(2;i,j\right)=f\left[u\left(2;i,j\right)\right]=f\left[C\left(1;i,j\right)\right]. \end{array}$$

In the C3 layer, similar to the C1 layer, each feature map of the S2 layer is convolved with a 5 × 5 convolution kernel to obtain 12 convolution feature maps with a size of 18 × 18. The C3 layer has 312 trainable parameters and 3886 neurons. The calculation formula is

(3) $$\begin{array}{c} \text{C}\left(3;i,j\right)=f\left[u\left(3;i,j\right)\right]=f\left[\prod_{i=1}^{6}S\left(2;i,j\right)\cdot k\left(3;i,j\right)-b\left(3;j\right)\right]. \end{array}$$

The S4 layer is a sampling layer composed of 12 feature maps. By sampling each feature map of the C3 layer with a sampling unit of 2 × 2, the same number of feature maps but with a reduced resolution of 9 × 9 can be obtained, and the calculation formula is

(4) $$\begin{array}{c} \text{S}\left(4;i,j\right)=f\left[u\left(4;i,j\right)\right]=f\left[C\left(3;i,j\right)\right]. \end{array}$$

Then, the feature maps extracted by the downsampling layers S2 and S4 are directly transferred to the fully connected layer F5 for fusion. The number of neurons in the fully connected layer nF5 is the sum of the number of neurons in the S2 layer nS2 and the number of neurons in the S4 layer nS4. There are a total of 3874 neurons. Through the above processing, each video frame is degraded into multiple single-pixel feature images, which are used as input for classification processing. The F5 layer is fully connected with the subsequent SVM multiclass behavior classifier.

### 4.3. Parameter Selection of Cross-Layer CNN-SVM Model

The choice of learning rate has an important influence on the training speed of the network. When the learning rate is larger, the adjustment range of the weights during the training process is larger, the convergence speed is faster, and the learning rate is higher. However, it may happen that the training process cannot be converged due to the instability of the network, and it may be Go over some weights that are close to optimization; the smaller the learning rate, the smaller the weight change, which can stably make the network approach the global optimal point and maintain the stability of the network, but it may fall into the local optimal region while learning convergence. Under normal circumstances, the learning speed of each neuron in the network is close by adjusting the learning rate. Because the local gradient of the neurons in the output layer is larger, the learning rate of the output layer is smaller; the learning rate of neurons with more neurons in the input layer is lower than the learning rate of neurons with fewer neurons. Generally, the learning rate is within the range of 0.01–0.76. For a more complex network, the learning rate is different for different positions of the error surface: in the flat area of the error surface, the learning rate is larger, while, in the area where the error surface is steep, the learning rate is smaller. To sum up, we read the literature, refer to related materials, and finally select the initial value of the learning rate of the cross-layer CNN-SVM as 1.

This article implements network initialization by assigning all weights and thresholds in the network to an initial value. When the selected initial value is in the flat area of the error surface, the convergence speed of the network training will slow down. Normally, the weights and thresholds of the network are initialized in a uniformly distributed form in a small area with a mean value.

For the Sigmoid activation function, since the gradient far away from the origin quickly tends to 0, the initialization of the network weights and thresholds mostly uses the decimal random average distribution method. It is hoped that more amplitude space can be adjusted for the neural network to improve learning speed. Since the Re Lu activation function used in this article has no similar problems, the initialization flexibility is relatively large, and random initialization can be used. Since the trained network weights and thresholds usually present a Gaussian distribution, we sample from a unit Gaussian distribution with a mean of 0 and a standard deviation of 1.

### 4.4. CNN Training and Learning

Before performing tasks, the cross-layer CNN-SVM model proposed in this paper needs to train the feature extraction model CNN. According to the characteristics of the cross-layer structure, the commonly used neural network training method, the backpropagation algorithm, is improved, and a training algorithm suitable for the cross-layer CNN in this article is designed.

Through the input behavior sample video, the error between the output vector and the reference vector is used to correct the parameters in the calculation process, so as to achieve the purpose of reducing the output error. Suppose that there are N training samples (*x*, *y*), the input of the network is denoted as *x*, the expected output is denoted as *y*, and the actual output is denoted as *o*. The objective function *E*_*N*_ is defined as the mean square error of all samples. The calculation formula is

(5) $$\begin{array}{c} E_{N}=0.5\prod_{l=1}^{n}\prod_{i=1}^{N}{\left(y_{i}-o_{i-1}\right)}^{2}. \end{array}$$

Then, the feedback transmission error *δ* of each layer in the network is calculated. The output layer and the upper layer neurons are fully connected, so the output layer error is equal to the difference between the actual output and the expected output multiplied by the partial derivative of the activation function with respect to the input vector. The calculation formula is

(6) $$\begin{array}{c} \delta \left(6;i\right)=\left|y_{i}-o_{i}\right|\times f^{\prime }\left[u\left(6;i-1\right)\right]. \end{array}$$

Since the convolutional layer and the sampling layer are alternately connected, the neuron in the convolutional layer is only connected to one neuron in the sampling layer. In order to calculate the error signal of the convolutional layer more conveniently and effectively, the error image of the sampling layer is internally filled with Kronecker product, and the dimension of the error of the sampling layer is consistent with the dimension of the output feature map of the convolutional layer through the upsampling process, and then the error calculation is performed on the convolutional layer. The upsampling function is defined as

(7) $$\begin{array}{c} {\text{up}}_{\lambda \cdot \Gamma }\left(x\right)=l_{\lambda \cdot \Gamma }⊗\left|x\right|. \end{array}$$

In the formula, *λ* × Γ is the multiple factor for downsampling, 1*λ* × Γ is a matrix with size *λ* × Γ and element value 1, and “⊗” represents the Kronecker product internal filling operation. The error calculation formula of the convolutional layer C3 is

(8) $$\begin{array}{c} \delta \left(3;i,j\right)={\left(\lambda \cdot \Gamma \right)}^{-1}\cdot \left[{\text{up}}_{\lambda \cdot \Gamma }\cdot \delta \left(4;i,j\right)\cdot f^{\prime }\left[u\left(3;i,j\right)\right]\right]. \end{array}$$

Because the downsampling layer S2 is connected to the downsampling layer S4 and the fully connected layer F5 at the same time, the error of S2 is equal to the sum of the errors of the S4 layer and the F5 layer. Like C3, the error calculation formula of the convolutional layer C1 is

(9) $$\begin{array}{c} \delta \left(1;i,j\right)={\left(\lambda \cdot \Gamma \right)}^{-1}\cdot \left[{\text{up}}_{\lambda \cdot \Gamma }\cdot \delta \left(1;i,j\right)\right]\cdot f^{\prime }\left[u\left(1;i,j\right)\right]. \end{array}$$

After the feedback transmission error of each layer is obtained, the weight partial derivative and the bias partial derivative are solved. Since the downsampling calculation method is simplified, only the convolutional layer and the output layer need to be calculated.

## 5. System Simulation Test

### 5.1. Attitude Detection and Attitude Control Function Test

During the system test, the nursing bed attitude detection and control circuit module is installed in the controller card slot under the nursing bed. Two high-precision angular displacement sensors are installed on the back support plate of the nursing bed and the connecting shaft between the leg support plate and the bed body. The serial port MPU6050 circuit module is attached to the central support plate of the nursing bed that cannot move freely. After that, the angular displacement sensor, MPU6050 module, and control handle are all connected to the attitude detection and control circuit module with signal lines. A signal line is used to connect the posture detection and control module with the monitoring center module, and the blood oxygen equipment, camera, and touch screen are, respectively, connected to the monitoring center module.

After the intelligent monitoring system is set up, we use the RS232 serial cable to connect the PC serial port and the debugging serial port of the monitoring center module. In the debugging state, the monitoring center module will transmit the issued control commands and received posture data to the PC in real time and use the serial debugging assistant to view the received data on the PC to monitor the system operation. When there is no control command, the monitoring center module will periodically send status query instructions to the attitude detection and control module, and the attitude detection and control module returns status data; when there is a control command, the monitoring center module first sends the control command and then sends the status query command.

**Figure 5:**
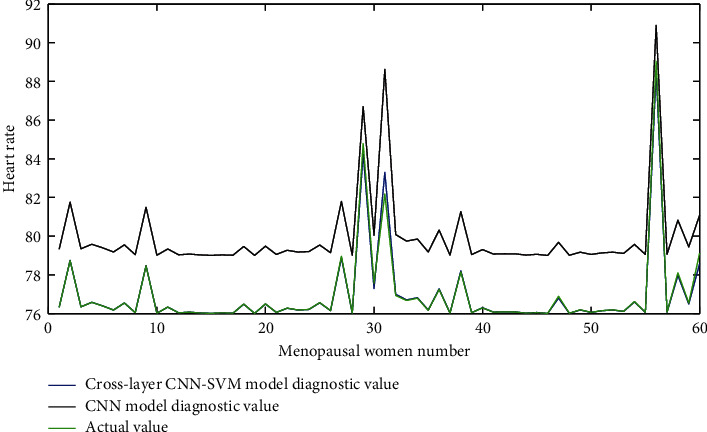
Heart rate data information when the health care bed for menopausal women is “folded back.”

**Figure 6:**
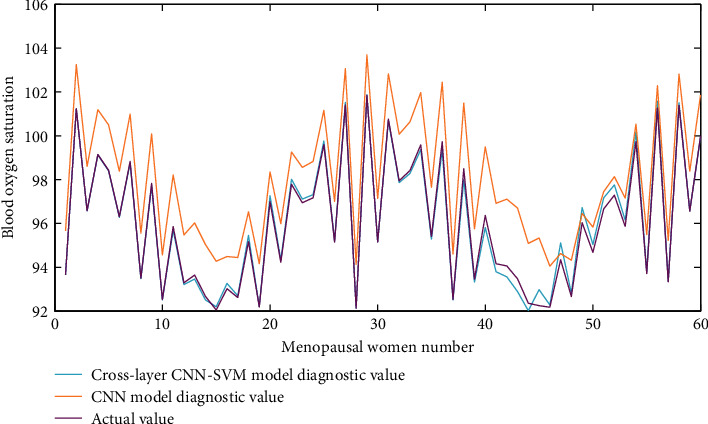
Blood oxygen saturation data information when the health care bed for menopausal women is “folded back.”

Figure 5 shows the heart rate data information when the menopausal women's health care bed “folds back.” The blood oxygen saturation data information when the menopausal women's health care bed “folds back” is shown in Figure 6. When the “back fold up” button is pressed, the monitoring center module sends the control command word “AB 83 AD” to the posture detection and control module and then immediately sends the query command word “EE 52 ED.” After the monitoring center module receives and processes the data, it displays the current posture angle of the back of the nursing bed and the current operation in progress on the monitoring center software interface.

**Figure 7:**
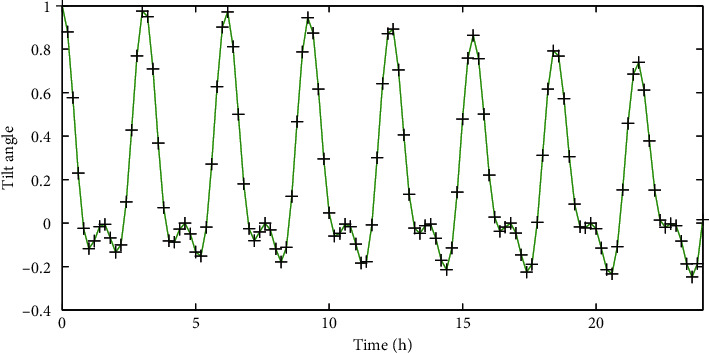
Display of abnormal posture information of the front and back tilt angle of the bed.

In order to test whether the system can accurately detect the abnormal posture of the nursing bed, when all parts of the nursing bed are kept horizontal, one side of the bed is manually raised to tilt the bed back and forth. Taking into account the system measurement error, even when the bed is level, there is a certain deviation in the measured angle. Therefore, it is assumed that the posture is abnormal when the current back tilt angle exceeds 5 degrees. In fact, when the system detects that the tilt angle has increased to 5 degrees, it will give a warning of abnormal attitude, which is within the allowable range of error. The 24 h front and rear tilt posture angle information display of the bed is shown in Figure 7.

In the above functional test process, through the process of observing the data received by the module, it shows that the monitoring center module correctly received the control command and sent it to the attitude detection and control module in time; the attitude detection and control module responded to the control command in a timely manner. After the monitoring center software receives the attitude data, it accurately calculates the attitude data and displays it in real time on the software interface. It shows that the system can indeed change and monitor the posture of the nursing bed quickly and accurately.

### 5.2. Timing Control Function Test of Health Care for Menopausal Women

**Figure 8:**
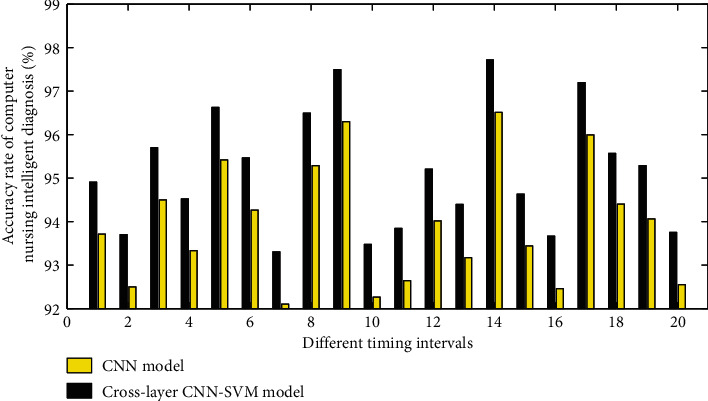
The accuracy of computer nursing intelligent diagnosis at different timing intervals.

The intelligent monitoring system allows the nursing staff to set the timing operation in advance, and the system will automatically control to change the posture of the nursing bed after the timing time is up.  Figure 8 shows the time consumption of computer nursing diagnosis at different timing intervals.

The system allows to set up three consecutive groups of timing operations, and each group can set a specific action time. The system will automatically control the various parts of the nursing bed to change their postures to reach the designated action angle after the timing of each set of operations is up. If only two sets of operations are set, the system can also be executed cyclically between the two sets of operations, so that the action of the nursing bed is regularized, and it is more convenient for the nursing work of the bedridden.

### 5.3. Computer Nursing Remote Diagnosis Function Test

The bottom layer of the Linux system transplanted on the monitoring center module sets the WIFI to automatically connect, but you need to manually set the name and password of the router that needs to be connected. After the modification is completed, it will be saved and exited, and the module will automatically connect to the wireless router. The development process of remote diagnosis client software is not the research content of this article. In order to reduce the workload of development and maintain the consistency of the monitoring system, the remote diagnosis terminal software interface adopts a design similar to that of the monitoring center software.

**Figure 9:**
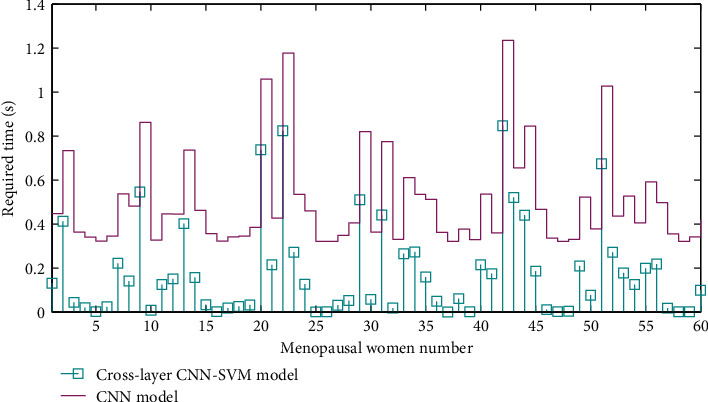
Time required for computer nursing remote diagnosis data processing.

Due to limited conditions, the client and the nursing bed intelligent monitoring system were in the same local area network during the test. After the connection is successful, the client will display a successful connection sign, and the monitoring center software interface will display the connection status and the client's IP address and other information. After the connection is successful, the monitoring center will automatically send the status data and monitoring video images of the nursing bed and menopausal women to the client. At this time, the remote client and the monitoring center software will simultaneously display the status information of the nursing bed and the bedridden. The time required for computer nursing remote diagnosis data processing is shown in Figure 9.

## 6. Conclusion

This paper studies the cross-layer CNN-SVM model and describes the overall plan of the behavioral intelligent diagnosis of care recipients and the specific implementation steps. The network model is analyzed in detail, the preprocessing methods of sample data are introduced, and the regularization of sample data is realized. By adopting a cross-layer structure, the limitations of menopausal women's behaviors such as unclear images are improved, and the structure and the model algorithm are described in detail. The three activation functions of Sigmoid function, Tanh function, and Re Lu function are analyzed and compared through experiments. The network parameters, including learning rate, network weights, and thresholds, are selected. The model training and learning process is analyzed, and the backpropagation algorithm process is described. The home care bed intelligent monitoring system designed in this paper can not only control the care bed to complete various posture changes, but also add the posture automatic detection function on this basis. The introduction of this function further improves the safety of the nursing bed, so that the nursing bed can realize autonomous control of posture changes. In addition, the system is equipped with a monitoring center component for the home care bed. Through the monitoring center, the posture of the care bed and the body condition information of the bedridden can be monitored in real time, video monitoring and remote diagnosis are realized, and the functions of the care bed are enriched. The home care bed intelligent monitoring system has been actually tested and used, and the function has achieved the expected effect, and the research task of the subject has been well completed. The system improves the safety of the nursing bed to a large extent, greatly enriches the functions of the nursing bed, can better realize the nursing operation of the care recipient, further reduces the burden of the nursing staff, and has certain practical application value.

## Data Availability

The data are available from the corresponding author upon request.
